# OMA orthology in 2024: improved prokaryote coverage, ancestral and extant GO enrichment, a revamped synteny viewer and more in the OMA Ecosystem

**DOI:** 10.1093/nar/gkad1020

**Published:** 2023-11-14

**Authors:** Adrian M Altenhoff, Alex Warwick Vesztrocy, Charles Bernard, Clement-Marie Train, Alina Nicheperovich, Silvia Prieto Baños, Irene Julca, David Moi, Yannis Nevers, Sina Majidian, Christophe Dessimoz, Natasha M Glover

**Affiliations:** SIB Swiss Institute of Bioinformatics, 1015 Lausanne, Switzerland; ETH Zurich, Computer Science, Universitätstr. 6, 8092 Zurich, Switzerland; SIB Swiss Institute of Bioinformatics, 1015 Lausanne, Switzerland; Department of Computational Biology, University of Lausanne, 1015 Lausanne, Switzerland; SIB Swiss Institute of Bioinformatics, 1015 Lausanne, Switzerland; Department of Computational Biology, University of Lausanne, 1015 Lausanne, Switzerland; Department of Computational Biology, University of Lausanne, 1015 Lausanne, Switzerland; Department of Computational Biology, University of Lausanne, 1015 Lausanne, Switzerland; SIB Swiss Institute of Bioinformatics, 1015 Lausanne, Switzerland; Department of Computational Biology, University of Lausanne, 1015 Lausanne, Switzerland; SIB Swiss Institute of Bioinformatics, 1015 Lausanne, Switzerland; Department of Computational Biology, University of Lausanne, 1015 Lausanne, Switzerland; SIB Swiss Institute of Bioinformatics, 1015 Lausanne, Switzerland; Department of Computational Biology, University of Lausanne, 1015 Lausanne, Switzerland; SIB Swiss Institute of Bioinformatics, 1015 Lausanne, Switzerland; Department of Computational Biology, University of Lausanne, 1015 Lausanne, Switzerland; SIB Swiss Institute of Bioinformatics, 1015 Lausanne, Switzerland; Department of Computational Biology, University of Lausanne, 1015 Lausanne, Switzerland; SIB Swiss Institute of Bioinformatics, 1015 Lausanne, Switzerland; Department of Computational Biology, University of Lausanne, 1015 Lausanne, Switzerland; SIB Swiss Institute of Bioinformatics, 1015 Lausanne, Switzerland; Department of Computational Biology, University of Lausanne, 1015 Lausanne, Switzerland

## Abstract

In this update paper, we present the latest developments in the OMA browser knowledgebase, which aims to provide high-quality orthology inferences and facilitate the study of gene families, genomes and their evolution. First, we discuss the addition of new species in the database, particularly an expanded representation of prokaryotic species. The OMA browser now offers Ancestral Genome pages and an Ancestral Gene Order viewer, allowing users to explore the evolutionary history and gene content of ancestral genomes. We also introduce a revamped Local Synteny Viewer to compare genomic neighborhoods across both extant and ancestral genomes. Hierarchical Orthologous Groups (HOGs) are now annotated with Gene Ontology annotations, and users can easily perform extant or ancestral GO enrichments. Finally, we recap new tools in the OMA Ecosystem, including OMAmer for proteome mapping, OMArk for proteome quality assessment, OMAMO for model organism selection and Read2Tree for phylogenetic species tree construction from reads. These new features provide exciting opportunities for orthology analysis and comparative genomics. OMA is accessible at https://omabrowser.org.

## Graphical Abstract

**Graphical Abstract:**
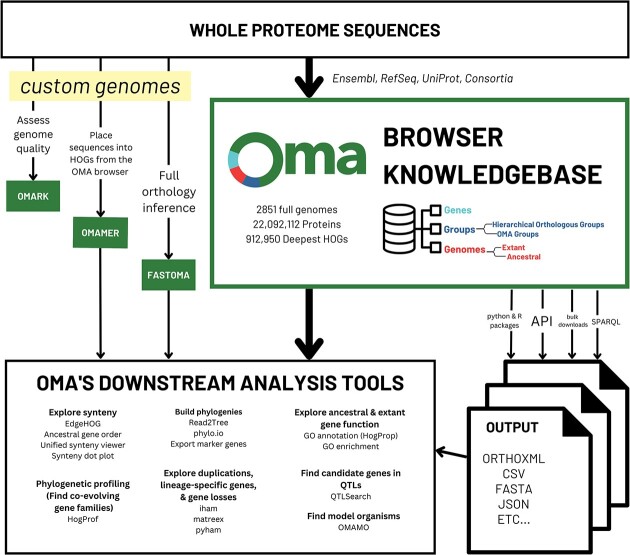

## Introduction

Genes which are related through speciation are called orthologs, as opposed to paralogs, which are related through duplication (1). This distinction is useful in a wide range of contexts, including phylogenetic tree inference, protein function prediction, or whole genome alignment (reviewed in (2)).

For more than 18 years, the OMA (‘Orthologous Matrix’) database has elucidated orthologs among complete genomes across the entire tree of life (3–7). In this update paper, we report on the most recent developments, including new and updated species, ancestral Gene Ontology annotations and synteny reconstruction, and an overview of the tools we provide to facilitate the use of the OMA knowledgebase.

## New and updated species—a better sampling of prokaryotic phylogenetic diversity

**Figure 1.**
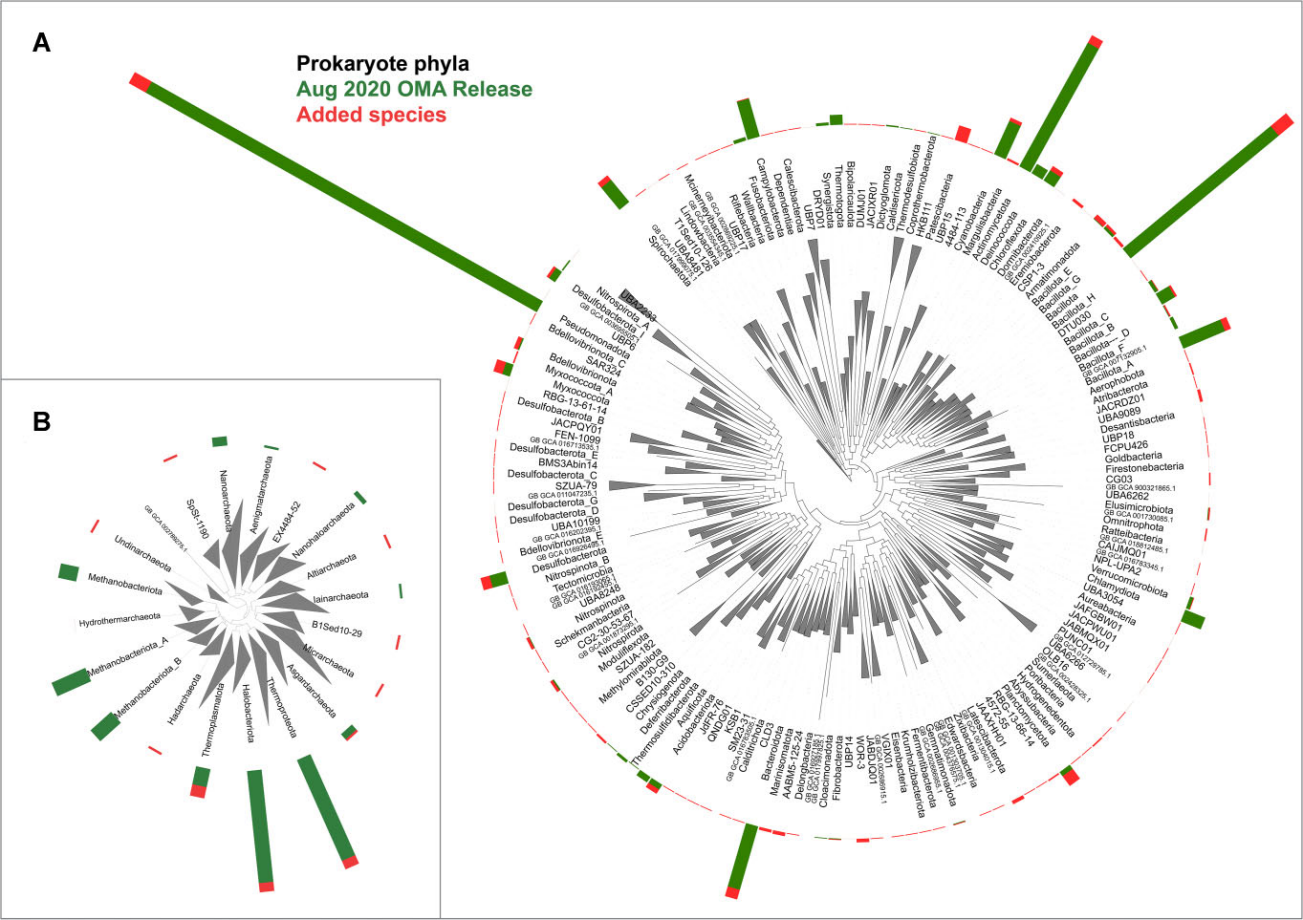
Improved coverage of prokaryotic diversity in the latest OMA browser release (July 2023). The two panels display the bacterial tree (**A**) and the archaeal tree (**B**) from GTDB release 207 (9), collapsed at the level of a GTDB phylum and labeled according to the updated nomenclature of prokaryotic phyla (11). Leaves with an assembly identifier label correspond to deep branching genomes without closely related genomes that are treated by GTDB as forming a phylum on their own. The height of each histogram bar is indicative of the proportion of the total number of bacterial (A) or archaeal (B) genomes available in OMA originating from the corresponding phylum. Green reflects the number of genomes in the Aug2020 OMA release, and red the additional genomes in the Jul 2023 release. Despite the bacteria coverage being heavily biased towards the Pseudomonadota (formerly Proteobacteria), Bacillota (formerly Firmicutes) and Actinomycetota (formerly Actinobacteria) phyla, with the updated coverage, almost all phyla in GTDB have at least one representative.

In this update, we’ve significantly broadened the representation of prokaryotic species within the OMA database. Since the last OMA paper, the number of bacteria species we include has risen from 1607 to 1965 in our latest release (+22%), and the number of archaea species from 152 to 173 (+14%; Figure 1). This new sampling aims to reflect the massive expansion of the described prokaryotic diversity recently enabled by metagenomics (8). Notably, we now include genomes from the novel Patescibacteria bacterial phylum (also known as the Candidate Phyla Radiation), as well as the DPANN and Asgardarchaeota archaeal lineages (8). This improved coverage of the prokaryotic diversity allows for a more comprehensive understanding of microbial genome evolution.

Our sampling strategy aims for a balanced representation, as we sought to include at least one species from each class in the Genome Taxonomy Database (GTDB (9)). The representative genome for each taxonomic class was chosen based on the completeness and contamination as assessed by CheckM (10), the length of contigs and the species’ importance within the class, reflected by the number of available genomes for this species.

Since the latest OMA paper we have also added 230 new Eukaryotic genomes (+47%). These additional genomes were selected to improve the sampling diversity. The species present in the OMA database can be found from the home page under *Explore* → *Species/Release information*. The prioritization of new and updated genomes is informed by our users, so we invite researchers to suggest new or updated genomes by filling in the following form: https://omabrowser.org/suggest.

## Ancestral genome gene content and gene order

The OMA database uses HOGs to model ancestral genomes corresponding to each internal node of OMA’s sampled Tree of Life. Conceptually, HOGs can be thought of as ancestral genes, as they encompass orthologs and paralogs descending from a common ancestral gene at a specific taxonomic level. Thus, the HOGs are proxies for ancestral genes in a common ancestor and the collection of HOGs at a given level are proxies for ancestral genomes.

**Figure 2.**
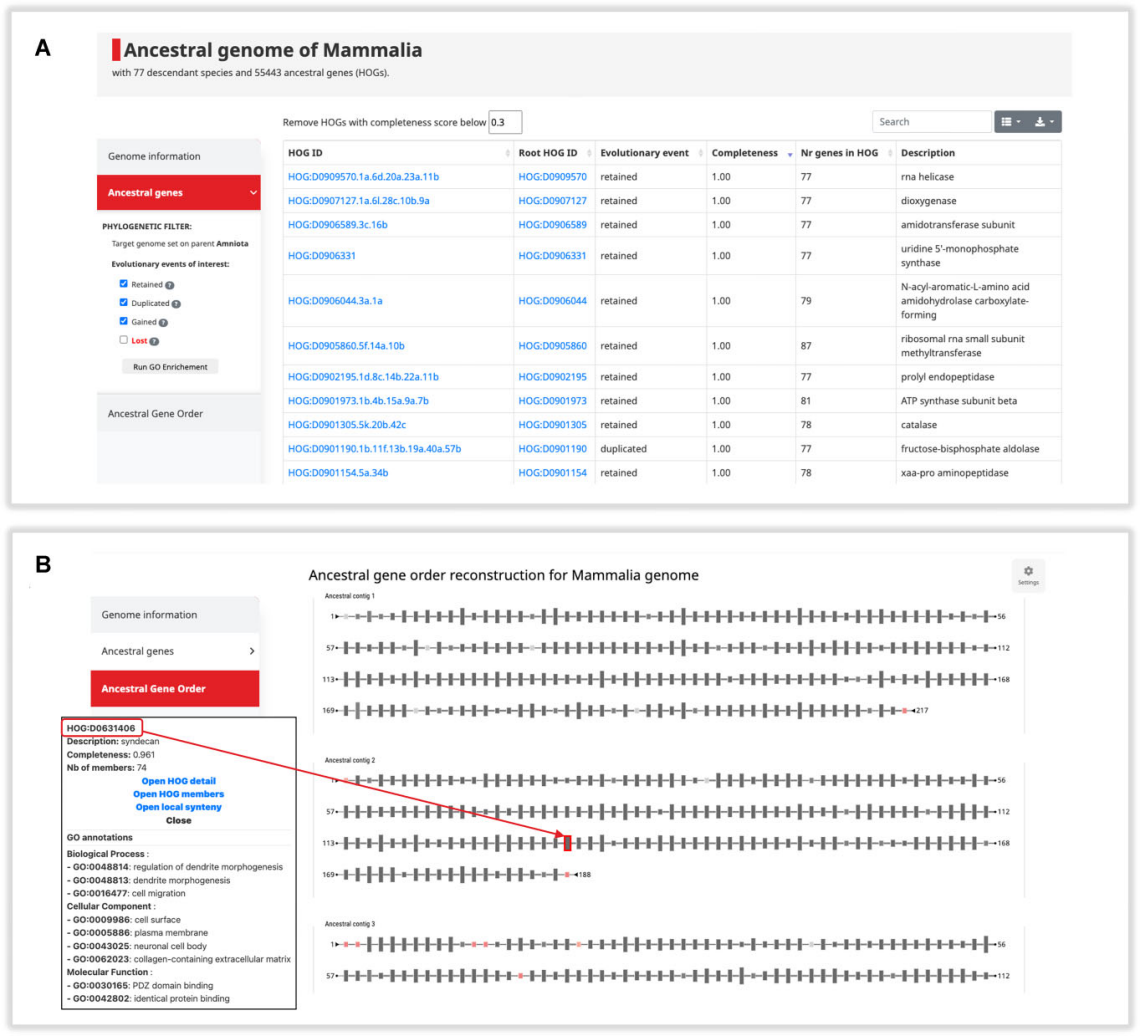
Ancestral Genome page for Mammalia. (**A**) Screenshot of the Ancestral Genes table. Here, each row is a HOG, i.e. ancestral gene, at the taxonomic level of interest. For each gene, the HOG identifier for that level and the HOG identifier of the entire gene family (‘Root HOG ID’) is shown. The ‘Evolutionary event’ column reflects if the gene was retained in a single copy, duplicated, or gained (originated) on the branch leading to this taxonomic level. These events can be filtered using the side menu. (**B**) Screenshot of the Ancestral Gene Order viewer. Here, the reconstructed gene order is shown: HOGs at that taxonomic level are represented as rectangles, connected based on evidence of gene order in the extant genomes. Node color indicates the Completeness Score (0 = red, 1 = dark grey), and node size is the number of extant genes in the HOG.

To facilitate ancestral genome exploration, we now offer a total of 1133 Ancestral Genome pages on the OMA browser (Figure 2A). Ancestral genomes can be accessed by searching for a specific taxa name or identifier, or by the *Explore* → *Quick access to* → *Extant and ancestral genomes*. These dedicated pages show all the HOGs specific to chosen taxonomic levels. From these pages, users can access details about that ancestral genome, including the number of descendant species found in the OMA database and the number of genes inferred to have existed in this common ancestor. The genes are displayed in a table by clicking on Ancestral genes in the left-hand menu. Recognizing that inferred counts can sometimes be influenced by HOG inference errors, particularly in the presence of highly divergent sequences, an option to filter for high-quality HOGs is provided. We define this ‘Completeness Score’ as the number of species included in the HOG divided by the total number of species in the clade, thus it ranges from 0 to 1 (default: 0.3). HOGs with a low Completeness Score could be dubious, as it would imply a high number of gene loss events.

HOGs offer insight into the evolutionary history of genes, thus the Ancestral Genome pages offer access to details concerning the evolutionary history of the HOGs present at that taxonomic level, which we compute with pyham (12). The left-hand menu displays the parental genome used to determine the evolutionary events that happened on the branch leading to the ancestor of interest. For example, if investigating the Mammalia genome, ‘Amniota’ is chosen as the most recent parental genome. The column ‘Evolutionary event’ in the Ancestral Genes table gives the status of the gene from the parental ancestor to the ancestor of interest. In this context, ‘retained’ denotes a gene's consistent presence as a single copy from the parent to the child. ‘Duplicated’ signifies that the gene arose due to a duplication event between the previous common ancestor and the ancestor of interest. In contrast, ‘gained’ signifies OMA’s inference that the gene emerged at the chosen taxonomic level (i.e. a root HOG). In the example in Figure 2A, the evolutionary events refer to what happened to the gene on the branch leading from Amniota to Mammalia. The evolutionary event displayed in the table can be filtered using the left-hand menu. We also give the option to display ‘Lost’ genes, which are genes present in the parental genome, but not in the ancestral genome of interest. These ‘phylostratigraphic’ gene pools hold potential for functional enrichment—an avenue of exploration detailed in the section ‘Ancestral and extant Gene Ontology enrichment analysis’.

As opposed to the previous version of OMA in which an ancestral genome was solely represented as a flat list of genes, the last update integrates ancestral gene order inferences to represent an ancestral genome as a collection of inferred contiguous regions of genes. An ancestral contig is depicted as a linear graph that can be interactively explored in our new ancestral gene order viewer by clicking on the ‘Ancestral Gene Order’ section of any Ancestral Genome page (Figure 2B). In this representation, a node corresponds to a HOG inferred to be a gene present in the ancestral genome, and each edge links two HOGs inferred by parsimony to be of closest proximity, with a weight equal to the number of supporting contexts in descendant extant genomes. Users can visualize the inferred contiguous regions of genes, ordered by decreasing length. Within each ancestral contig, hovering over an edge will display its weight, while clicking on a node will display the identifier of the corresponding HOG, its functional description and the list of associated GO terms ranked by information content (see ‘HOG (ancestral gene) annotation’ section below).

This additional level of resolution introduced with ancestral gene order inferences unlocks two key applications. First, it makes it possible to track the chain of genomic rearrangements that occurred during the evolution of lineages, with opportunities to flag past rearrangements that correlate with adaptation (13–15). Second, the reconstructed gene orders allow for identifying conserved genomic neighborhoods, which can be indicative of functional coupling between adjacent genes (16,17). Within any clade of interest, including clades as old as Eukaryota, Bacteria or Archaea, the corresponding ancestral contiguous regions allow for ‘guilt by association’ searches (18–20). This method has proven to be remarkably successful at identifying novel candidate operons, metabolic pathways, macromolecular systems, biosynthetic gene clusters and prokaryotic defense systems (21–24).

## Ancestral and extant local synteny viewer

**Figure 3.**
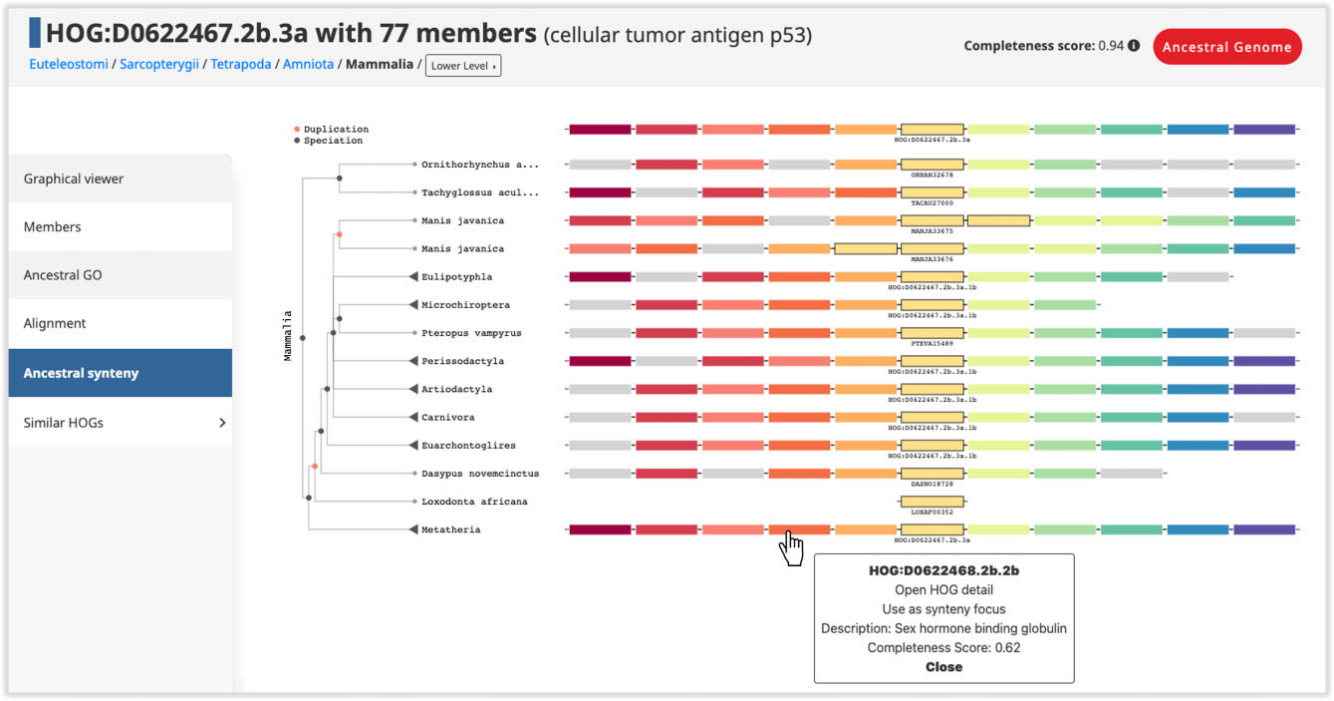
Ancestral and extant unified local synteny viewer. The boxes represent extant or ancestral genes, with genes of the same color related by homology, i.e. found in the same HOG at the taxonomic level of interest. The focal HOG (or extant gene) and its homologs are outlined in black, with their names shown below. In this example, the focal HOG displayed is the cellular tumor antigen p53 at the Mammalia level. The top row is the reconstructed genomic neighborhood of this gene in the Mammalian common ancestor. The reconciled gene tree is displayed to the left, with the genomic neighborhood of each ancestral or extant genome shown next to it. Duplication nodes are shown in red. The viewer is interactive in that users can collapse nodes on the tree to display the genomic neighborhood in the ancestor corresponding to that node. Clicking on an ancestral gene displays the HOG ID and the functional description of the HOG, and clicking on an extant gene displays the identifiers, sequence length, chromosome, description and the HOG it belongs to.

In this update, we took advantage of the inferred ancestral contiguous regions of genes to propose a new unified, gene-centric local synteny viewer. For any query HOG or extant gene, it is now possible to visualize the multiple alignment of its inferred genomic neighborhood at the taxonomic level of investigation with that of all its descendant orthologs and paralogs in intermediate and extant descendant genomes (Figure 3). This kind of unified local synteny viewer enabling interactive comparison of both extant and ancestral genomic neighborhoods in a phylogeny-aware context has been provided by specialized databases—notably the Yeast Gene Order Browser (25) and Genomicus (26), but the scale and depth of our reconstructions is unprecedented.

At the top of the view, the reference ancestral neighborhood of the query HOG is shown. It is composed of the central query HOG and up to five inferred flanking HOGs in both directions. To the left of the view, the reconciled gene tree derived from the query HOG is shown. The gene neighborhoods of extant genes in the query HOG are displayed to the right, corresponding to the leaves of the gene tree. Genes which descended from the same HOG have the same color, and genes which are colored grey indicate no inferred homology to the genes in the query HOG neighborhood. These features allow for visualizing and investigating the conservation of the genomic neighborhoods of a gene family in a phylogeny-aware context.

Users can collapse clades of extant genes. Upon collapsing, the collapsed clade of extant genes becomes represented by the inferred neighborhood of their last common ancestral gene (i.e. the HOG). This option provides a way to compare the genomic neighborhood of a gene in a more ancient ancestor to the descendant genes in intermediate ancestors or extant species. Users can continue to collapse older clades, further customizing the display. In addition to allowing for a more compressed visualization, collapsing nodes can also be practical in case of large query HOGs or HOGs that have an overrepresentation of closely related species in that clade, saturating the view.

## Ancestral and extant Gene Ontology annotation and functional enrichment analysis

### HOG (ancestral gene) annotation

The hierarchical nature of the HOG structure facilitates exploration of gene family and species evolution. By adding functional information to the HOGs, we can gain further insight into the functional evolution of genes and genomes, and track when specific functions appeared throughout the Tree of Life. We now annotate the HOGs at every level in the OMA taxonomy with Gene Ontology (GO) terms. Using the framework of the HOGs and the HOGProp method (27), we parsimoniously propagated GO terms from extant genes to the HOGs that encompass them. Thus, HOGProp yields ancestral genes annotated with GO terms.

In the latest release, we started with 486 837 274 GO annotations for 14 107 684 (63.86%) proteins over all extant species in OMA, and used this information to annotate 26 512 830 (88.14%) HOGs with at least 1 GO term, across 507 406 (55.58%) root-level HOGs. Of the root-level HOGs with GO annotations, the average number of GO terms is 27. This represents functional annotation for a significant proportion of the ancestral genomes at key taxonomic levels (Table 1).

**Table 1.** GO annotation coverage

Ancestral level	No. HOGs annotated with GO term	No. HOGs in total	Percentage of HOGs annotated with GO term
Last Universal Common Ancestor (LUCA)	9418	12 995	72.5%
Bacteria	29 163	49 142	59.3%
Archaea	7851	10 152	77.3%
Eukaryota	31 193	40 760	76.5%
Fungi	36 261	41 017	88.4%
Viridiplantae	37 464	42 339	88.5%
Protostomia	63 491	77 876	81.5%
Deuterostomia	49 909	59 484	83.9%
Metazoa	49 145	56 762	86.6%
Vertebrata	53 579	61 808	86.7%
Primates	35 635	38 457	92.7%

Examples of key clades in the OMA database, the total number of HOGs at that taxonomic level, and the number/percentage of HOGs annotated with at least 1 GO term. Note that the number of HOGs displayed in the table are unfiltered for HOG quality, thus they may not reflect the true number of ancestral genes.

Ancestral GO annotations are available from the HOG Group pages on the left-hand menu.

### Ancestral and extant Gene Ontology enrichment analysis

Knowing the functions of ancestral genes allows for functional enrichment analysis. Traditionally performed on extant genomes, this analysis identifies functions that are over-represented in a study set (foreground) compared to the population (background).

Users can now carry out Gene Ontology enrichment analyses (GOEA) for sets of both extant genes or ancestral genes using the OMA browser. Extant gene GO enrichment is carried out as described in (28), where the study set is a user-defined set of extant genes, and the population is all genes in the extant genome of interest. For our newly-introduced ancestral gene GO enrichment feature, we exploit the HOG GO annotations by considering a study set of user-defined HOGs at a given taxonomic level, compared to the population of all HOGs defined at a given taxonomic level. Each GO term also implies its parental terms (29), so GO annotations are typically stored in a non-redundant manner, as a set of the most specific terms necessary to reconstruct the annotation. After propagating the more general GO terms for each member of the analysis, uncorrected *P*-values for overrepresentation of the study set compared to the population are generated using Fisher's exact test. Corrected *P*-values are obtained using either Bonferroni correction or the Benjamini-Hochberg procedure. To our knowledge, this method is the first instance of ancestral gene enrichment analysis available on a user-friendly browser.

**Figure 4.**
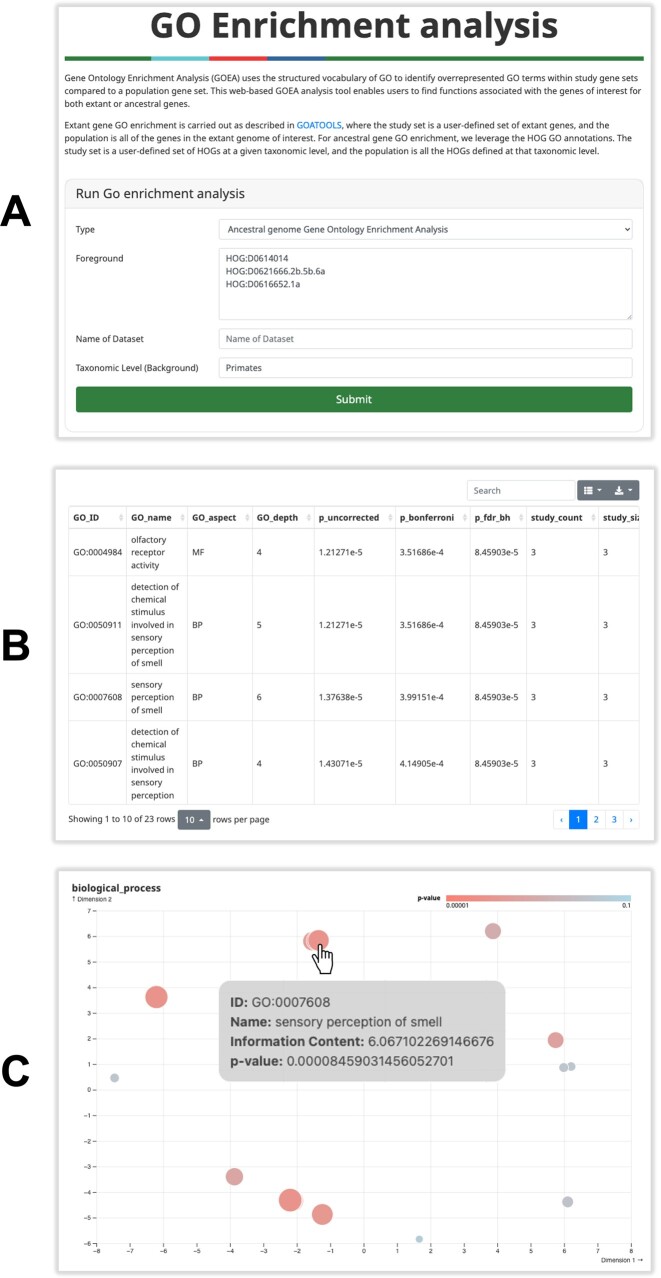
Extant and ancestral Gene Ontology enrichment analysis. (**A**) User interface to submit a study set of genes. (**B**) The resulting table displays the GO terms over-represented in the study set with a Benjamini-Hochberg FDR corrected *P*-value ≤0.05 Notably, users have access to the study and population sets, as well as the entries in the study set annotated with the GO term. (**C**) The Biological Process output plot showing enriched GO terms. Here, each bubble represents an enriched GO term, with the *P*-value indicated by color. The size of the bubble is proportional to the information content. Mousing over the bubbles gives the GO name, GO identifier, corrected *P*-value and information content. (Not shown: CC and MF.)

To enable users to perform GO enrichments on their own sets of ancestral or extant genes of interest, we offer both a browser interface and API functionality. The interface is straightforward: select the analysis type (Extant or Ancestral) and specify a study set of genes (Figure 4A). The input must be a list of identifiers separated by spaces, tabs, commas, or new lines. In the case of extant GO enrichment, OMA identifiers or other cross-reference ids are accepted; for ancestral GO enrichment, HOG identifiers from a specific taxonomic level are necessary (e.g. HOG:D0639603.1b) and must be indicated. For extant enrichments, OMA will identify the appropriate taxonomic level and report it in the output.

Upon computation, the results of the GO enrichment are displayed (Figure 4B, C). A table lists the enriched GO terms, featuring the Gene Ontology unique identifier, GO term name, which one of the three sub-ontologies that the term belongs to (MF: Molecular Function, BP: Biological Process, CC: Cellular Component), the uncorrected *P*-value as determined by Fisher's exact test, the *P*-value corrected for multiple testing using the Bonferroni and Benjamini–Hochberg procedure, the number, ratio and proportion of genes in the study set and population which are annotated with the GO term, and finally the fold-change between the study set and population.

We also provide up to three plots to summarize the significant GO terms for the MF, BP, and CC categories. Here, as in REVIGO and GO-Figure (30,31), the two dimensions represent a semantic space for the significant GO terms, resulting from a multi-dimensional scaling of the pairwise simRel semantic similarity measures (32). GO terms will appear closer to each other the more semantically similar they are. The bubble colors denote corrected *P*-values and the bubble size reflects the information content (33). Bubbles provide term and enrichment details upon hover. Full results including the table, plots, study set and population set (including the taxonomic level if inferred) can be downloaded in compressed files.

The GO enrichment analysis form can be accessed from the home page (*Tools* → *GO enrichment analysis*) or from an Extant or Ancestral Genomes page. From an Ancestral Genome page, GO enrichments can be performed on subsets of genomes by selecting phylostratigraphy categories (retained, duplicated, lost, or gained).

## Improved search engine

We have made significant enhancements to the OMA browser's search engine, resulting in a less ambiguous and more effective searching experience. This improvement centers on providing contextual understanding to query terms, which in turn leads to faster and more accurate search results.

This optimization is driven by the indexing of data such as the gene or HOG identifiers. The revamped search engine is built upon a token-based framework that relies on visual cues and logical associations. Users initiate a search by entering a query into the search field and pressing Space or Enter, generating a distinct token. These tokens consist of two parts: a prefix (field) that guides the search, and the query itself.

Tokens can be of different types, each representing a specific category: Gene, HOG, OMA Group, or Taxon. The prefixes of the tokens define the category that should be associated with the corresponding query term. For example, if a user inputs the token [go:4225], the search engine will look for genes in the OMA database that are annotated with the GO:0004225 Gene Ontology term. Without a token, ‘4225’ could refer to a taxon, HOG, or gene identifier, resulting in slower and more ambiguous searches.

Multi-word queries are supported if users enclose them in quotation marks (e.g. [species:‘homo sapiens’]). Editing queries is possible by clicking on them to modify the input field. Users can select and adjust prefixes by clicking on the dropdown icon to select a different one. Additionally, if one starts typing a query without hitting Enter or Space, OMA will automatically suggest identifiers after a few seconds, streamlining the search process.

Combining different tokens opens up possibilities for advanced searches. For instance, a search input like [hog:60627 species:HUMAN] would yield human genes found in HOG:606207.

## New applications and tools in the OMA Ecosystem

The OMA Ecosystem is the collection of tools, software, data downloads, interactive visualizations and other resources, which are associated with the OMA knowledgebase. The main entry point is the OMA browser. In this section, we highlight some tools which are recent editions to the OMA Ecosystem.

### OMAmer: mapping proteomes to existing HOGs

The OMA database contains HOGs with species covering a wide diversity of taxa. However, researchers are often interested in finding orthologs for proteins in other species. The OMAmer (34) software offers the possibility to quickly place protein sequences into existing HOGs from the OMA database.

OMAmer is a tool that helps identify orthologs and paralogs more effectively compared to sequence similarity-based methods. The limitation of naive methods is that the closest sequence may not necessarily belong to the same subfamily due to differences in evolutionary rates across the tree, among other complications (34). OMAmer overcomes this issue by using evolutionarily-informed *k*-mers for alignment-free mapping to HOGs. One of the key advantages of OMAmer is its ability to map not only to root HOGs (gene families), but also to the correct subfamilies. This level of delineation is crucial when studying the functional divergence of paralogs. OMAmer has demonstrated higher accuracy than approaches merely based on maximizing sequence similarity (35).

OMAmer users can quickly identify the most likely HOG for one or multiple proteins of interest. The OMA Browser provides an asynchronous tool that allows users to upload their custom proteomes and map them to HOGs. This tool has replaced the previous implementation of Fast Mapping, which was not based on OMAmer. The user can access the tool from the home page → *Tools* → *Fast Mapping with OMAmer*.

Alternatively, users can perform OMAmer searches locally, by using the software available at https://github.com/DessimozLab/omamer. A relevant OMAmer database is required, for which we now offer four precomputed OMAmer databases at each new release of the OMA database: one global database (LUCA.h5) and three clade-specific databases for more targeted searches. These OMAmer databases are available in the *Download* → *Current release* of the Browser and enable the processing of entire proteomes in minutes, allowing users to perform phylogenomics analyses without the computational cost of direct orthology calling. This is an option for users who would like to quickly and easily benefit from OMA’s wealth of precomputed data for their dataset.

By utilizing OMAmer, users can then explore the available information for the identified gene family in the OMA browser or export the sequence data for further analysis. This streamlined process makes it easier for researchers to analyze their custom proteomes and gain insights into orthologs and paralogs.

### OMArk: quality assessment of proteomes

OMArk is a new tool for quality assessment of proteomes, which can estimate not only the completeness, but also the prevalence of fragments, contamination and dubious gene models. OMArk uses OMAmer to place sequences rapidly into HOGs in the OMA database and then exploits the evolutionary information of the HOGs at that taxonomic level (i.e. presence of single-copy or duplicated genes) (36). Comparing user-defined proteomes to that which is expected, based on the OMA database, allows for evolutionarily-informed quality assessment. OMArk additionally identifies likely contamination within proteomes.

With the regular addition of high-quality and taxonomically diverse genomes to the OMA browser, we expect the resolution of OMArk estimates to improve with each new release. Since the November 2022 release, we now use OMArk quality assessments to update the OMA Browser by replacing lower quality proteomes with higher quality ones where available and to improve taxonomic diversity. This will continue to be part of our process for proteome selection and updates in the future, in combination with community requests.

A few examples of decisions that were influenced by OMArk in the latest release are as follows: we removed *Drosophila biarmipes* from our dataset due to contamination by *Saccharomyces cerevisiae* sequences. We added new birds (*Haliaeetus leucocephalus, Falco tinnunculus, Corvus moneduloides, Tyto alba*) and butterflies (*Vanessa tameamea* and *Papillo machao*), guided by OMArk quality metrics. In the latest release, we switched to the RefSeq annotation of the chicken genome and updated the tardigrade, *Hypsibius dujardini*, also guided by OMArk quality metrics.

With the increase in genomic data, OMArk will continue to help improve the OMA Browser dataset by improving orthology calling and other downstream analyses, which first depend on the quality of the underlying annotation data. OMArk is available at https://github.com/DessimozLab/OMArk and online at https://omark.omabrowser.org.

### OMAMO: selecting the best model organisms for a biological process of interest

The conservation of pathways across species enables researchers to use non-human organisms as models. However, the most widely-used model species include higher animals such as mice and zebrafish, which is costly, time-consuming, and which requires ethical consideration. These problems highlight the need for identifying less complex model species. Existing tools that focus on gene conservation across species for identifying model organisms only consider a very sparse set of single-cell species or none at all. To address these issues, we developed ‘Orthologous Matrix and Model Organisms’ (OMAMO) (37), a software tool that is integrated into the OMA browser. It identifies the most suitable less complex organism for research, given a biological process of interest. The OMAMO algorithm relies on the fact that orthologous genes tend to be functionally conserved and have similar expression patterns, unlike other types of homologs (38). It uses GO annotations of orthologous species-human pairs to estimate the functional similarity of genes in a given biological process, thereby providing a pathway-oriented approach for model organism search. The software can be used through the OMA browser (https://omabrowser.org/oma/omamo/search/) by searching a biological process GO term.

### Read2Tree: building phylogenetic species trees from reads

Phylogenetic trees play a crucial role in various contexts, but constructing high-quality trees remains highly challenging due to the complex multi-step process involved in state-of-the-art pipelines (39).

Read2Tree is a fast approach for phylogeny inference that avoids the computationally costly steps of genome assembly, annotation, sequence comparison and orthology inference. Read2Tree utilizes OMA marker genes, i.e. orthologous groups which only contain genes orthologous to each other, on which the input raw sequencing reads are mapped. It outputs a multiple sequence alignment and the species tree (39). Of note, Read2Tree is much faster (10-100x) compared to the conventional approaches with similar accuracy, or in some cases even more accurate. As opposed to assembly-based approaches which need complementary sequencing technologies with high coverage, Read2Tree works well with as low as 0.2x sequencing coverage with either short or long data (Illumina, PacBio, Oxford Nanopore). Read2Tree is available at https://github.com/DessimozLab/read2tree.

## Data Availability

OMA data are available in various formats, including the interactive website, flat files, RDF, REST API, R and Python libraries. OMA is licensed under a Creative Commons Attribution License CC-BY 4.0. The underlying sequences and annotations may be subject to third-party constraints. Users of the data are solely responsible for establishing the nature of and complying with any such intellectual property restrictions.

## References

[1] Fitch W.M. Distinguishing homologous from analogous proteins. Syst. Zool. 1970; 19:99–113. 5449325

[2] Glover N. , Dessimoz C., Ebersberger I., Forslund S.K., Gabaldón T., Huerta-Cepas J., Martin M.-J., Muffato M., Patricio M., Pereira C. et al. Advances and applications in the quest for orthologs. Mol. Biol. Evol. 2019; 36:2157–2164. 31241141 10.1093/molbev/msz150 PMC6759064

[3] Dessimoz C. , Cannarozzi G., Gil M., Margadant D., Roth A., Schneider A., Gonnet G McLysaght A. , Huson D.H. OMA, A comprehensive, automated project for the identification of orthologs from complete genome data: introduction and first achievements. RECOMB 2005 Workshop on Comparative Genomics. 2005; Springer-Verlag 61–72.

[4] Altenhoff A.M. , Schneider A., Gonnet G.H., Dessimoz C. OMA 2011: orthology inference among 1000 complete genomes. Nucleic Acids Res. 2011; 39:D289–D294. 21113020 10.1093/nar/gkq1238 PMC3013747

[5] Altenhoff A.M. , Škunca N., Glover N., Train C.-M., Sueki A., Piližota I., Gori K., Tomiczek B., Müller S., Redestig H. et al. The OMA orthology database in 2015: function predictions, better plant support, synteny view and other improvements. Nucleic Acids Res. 2015; 43:D240–D249. 25399418 10.1093/nar/gku1158 PMC4383958

[6] Altenhoff A.M. , Glover N.M., Train C.-M., Kaleb K., Warwick Vesztrocy A., Dylus D., de Farias T.M., Zile K., Stevenson C., Long J. et al. The OMA orthology database in 2018: retrieving evolutionary relationships among all domains of life through richer web and programmatic interfaces. Nucleic Acids Res. 2018; 46:D477–D485. 29106550 10.1093/nar/gkx1019 PMC5753216

[7] Altenhoff A.M. , Train C.-M., Gilbert K.J., Mediratta I., Mendes de Farias T., Moi D., Nevers Y., Radoykova H.-S., Rossier V., Warwick Vesztrocy A. et al. OMA orthology in 2021: website overhaul, conserved isoforms, ancestral gene order and more. Nucleic Acids Res. 2021; 49:D373–D379. 33174605 10.1093/nar/gkaa1007 PMC7779010

[8] Hug L.A. , Baker B.J., Anantharaman K., Brown C.T., Probst A.J., Castelle C.J., Butterfield C.N., Hernsdorf A.W., Amano Y., Ise K. et al. A new view of the tree of life. Nat. Microbiol. 2016; 1:16048. 27572647 10.1038/nmicrobiol.2016.48

[9] Parks D.H. , Chuvochina M., Rinke C., Mussig A.J., Chaumeil P.-A., Hugenholtz P. GTDB: an ongoing census of bacterial and archaeal diversity through a phylogenetically consistent, rank normalized and complete genome-based taxonomy. Nucleic Acids Res. 2022; 50:D785–D794. 34520557 10.1093/nar/gkab776 PMC8728215

[10] Parks D.H. , Imelfort M., Skennerton C.T., Hugenholtz P., Tyson G.W. CheckM: assessing the quality of microbial genomes recovered from isolates, single cells, and metagenomes. Genome Res. 2015; 25:1043–1055. 25977477 10.1101/gr.186072.114 PMC4484387

[11] Oren A. , Garrity G.M. Valid publication of the names of forty-two phyla of prokaryotes. Int. J. Syst. Evol. Microbiol. 2021; 71:e005056. 10.1099/ijsem.0.005056 34694987

[12] Train C.-M. , Pignatelli M., Altenhoff A., Dessimoz C. iHam and pyHam: visualizing and processing hierarchical orthologous groups. Bioinformatics. 2019; 35:2504–2506. 30508066 10.1093/bioinformatics/bty994 PMC6612847

[13] Kim J. , Farré M., Auvil L., Capitanu B., Larkin D.M., Ma J., Lewin H.A. Reconstruction and evolutionary history of eutherian chromosomes. Proc. Natl. Acad. Sci. U.S.A. 2017; 114:E5379–E5388. 28630326 10.1073/pnas.1702012114 PMC5502614

[14] Duchemin W. , Anselmetti Y., Patterson M., Ponty Y., Bérard S., Chauve C., Scornavacca C., Daubin V., Tannier E. DeCoSTAR: reconstructing the ancestral organization of genes or genomes using reconciled phylogenies. Genome Biol. Evol. 2017; 9:1312–1319. 28402423 10.1093/gbe/evx069 PMC5441342

[15] Muffato M. , Louis A., Nguyen N.T.T., Lucas J., Berthelot C., Roest Crollius H. Reconstruction of hundreds of reference ancestral genomes across the eukaryotic kingdom. Nat. Ecol. Evol. 2023; 7:355–366. 36646945 10.1038/s41559-022-01956-z PMC9998269

[16] Trowsdale J. The gentle art of gene arrangement: the meaning of gene clusters. Genome Biol. 2002; 3:COMMENT2002. 11897017 10.1186/gb-2002-3-3-comment2002 PMC139018

[17] Marcet-Houben M. , Collado-Cala I., Fuentes-Palacios D., Gómez A.D., Molina M., Garisoain-Zafra A., Chorostecki U., Gabaldón T. EvolClustDB: exploring eukaryotic gene clusters with evolutionarily conserved genomic neighbourhoods. J. Mol. Biol. 2023; 435:e168013. 10.1016/j.jmb.2023.168013 36806474

[18] Aravind L. Guilt by association: contextual information in genome analysis. Genome Res. 2000; 10:1074–1077. 10958625 10.1101/gr.10.8.1074

[19] Galperin M.Y. , Koonin E.V. Who's your neighbor? New computational approaches for functional genomics. Nat. Biotechnol. 2000; 18:609–613. 10835597 10.1038/76443

[20] Overbeek R. , Fonstein M., D’Souza M., Pusch G.D., Maltsev N The use of gene clusters to infer functional coupling. Proc. Natl. Acad. Sci. U.S.A. 1999; 96:2896–2901. 10077608 10.1073/pnas.96.6.2896 PMC15866

[21] Moreno-Hagelsieb G. , Collado-Vides J. A powerful non-homology method for the prediction of operons in prokaryotes. Bioinformatics. 2002; 18:S329–S36. 12169563 10.1093/bioinformatics/18.suppl_1.s329

[22] Abby S.S. , Néron B., Ménager H., Touchon M., Rocha E.P.C. MacSyFinder: a program to mine genomes for molecular systems with an application to CRISPR-Cas systems. PLoS One. 2014; 9:e110726. 25330359 10.1371/journal.pone.0110726 PMC4201578

[23] Kautsar S.A. , Blin K., Shaw S., Weber T., Medema M.H. BiG-FAM: the biosynthetic gene cluster families database. Nucleic Acids Res. 2021; 49:D490–D497. 33010170 10.1093/nar/gkaa812 PMC7778980

[24] Doron S. , Melamed S., Ofir G., Leavitt A., Lopatina A., Keren M., Amitai G., Sorek R. Systematic discovery of antiphage defense systems in the microbial pangenome. Science. 2018; 359:eaar4120. 29371424 10.1126/science.aar4120 PMC6387622

[25] Byrne K.P. , Wolfe K.H. The Yeast Gene order browser: combining curated homology and syntenic context reveals gene fate in polyploid species. Genome Res. 2005; 15:1456–1461. 16169922 10.1101/gr.3672305 PMC1240090

[26] Nguyen N.T.T. , Vincens P., Dufayard J.F., Roest Crollius H., Louis A. Genomicus in 2022: comparative tools for thousands of genomes and reconstructed ancestors. Nucleic Acids Res. 2022; 50:D1025–D1031. 34792170 10.1093/nar/gkab1091 PMC8728260

[27] Warwick Vesztrocy A. , Dessimoz C., Redestig H. Prioritising candidate genes causing QTL using hierarchical orthologous groups. Bioinformatics. 2018; 34:i612–i619. 30423067 10.1093/bioinformatics/bty615 PMC6129274

[28] Klopfenstein D.V. , Zhang L., Pedersen B.S., Ramírez F., Warwick Vesztrocy A., Naldi A., Mungall C.J., Yunes J.M., Botvinnik O., Weigel M. et al. GOATOOLS: a Python library for gene ontology analyses. Sci. Rep. 2018; 8:10872. 30022098 10.1038/s41598-018-28948-z PMC6052049

[29] Hastings J. Dessimoz C. , Škunca N. Primer on Ontologies. The Gene Ontology Handbook. 2017; NY Springer New York 3–13.

[30] Supek F. , Bošnjak M., Škunca N., Šmuc T. REVIGO summarizes and visualizes long lists of gene ontology terms. PLoS One. 2011; 6:e21800. 21789182 10.1371/journal.pone.0021800 PMC3138752

[31] Reijnders M.J.M.F. , Waterhouse R.M. Summary visualizations of gene ontology terms with GO-figure. Front. Bioinformatics. 2021; 1:6. 10.3389/fbinf.2021.638255 PMC9581009 36303779

[32] Schlicker A. , Domingues F.S., Rahnenführer J., Lengauer T. A new measure for functional similarity of gene products based on Gene Ontology. BMC Bioinf. 2006; 7:302. 10.1186/1471-2105-7-302 PMC1559652 16776819

[33] Liu W. , Liu J., Rajapakse J.C. Gene ontology enrichment improves performances of functional similarity of genes. Sci. Rep. 2018; 8:12100. 30108262 10.1038/s41598-018-30455-0 PMC6092333

[34] Rossier V. , Vesztrocy A.W., Robinson-Rechavi M., Dessimoz C. OMAmer: tree-driven and alignment-free protein assignment to subfamilies outperforms closest sequence approaches. Bioinformatics. 2021; 37:2866–2873. 33787851 10.1093/bioinformatics/btab219 PMC8479680

[35] Buchfink B. , Reuter K., Drost H.-G. Sensitive protein alignments at tree-of-life scale using DIAMOND. Nat. Methods. 2021; 18:366–368. 33828273 10.1038/s41592-021-01101-x PMC8026399

[36] Nevers Y. , Rossier V., Train C.M., Altenhoff A., Dessimoz C., Glover N. Multifaceted quality assessment of gene repertoire annotation with OMArk. 2022; bioRxiv doi:28 November 2022, preprint: not peer reviewed 10.1101/2022.11.25.517970. PMC11738984 38383603

[37] Nicheperovich A. , Altenhoff A.M., Dessimoz C., Majidian S. OMAMO: orthology-based alternative model organism selection. Bioinformatics. 2022; 38:2965–2966. 35561194 10.1093/bioinformatics/btac163 PMC9113245

[38] Zheng-Bradley X. , Rung J., Parkinson H., Brazma A. Large scale comparison of global gene expression patterns in human and mouse. Genome Biol. 2010; 11:R124. 21182765 10.1186/gb-2010-11-12-r124 PMC3046484

[39] Dylus D. , Altenhoff A., Majidian S., Sedlazeck F.J., Dessimoz C. Inference of phylogenetic trees directly from raw sequencing reads using Read2Tree. Nat. Biotechnol. 2023; 10.1038/s41587-023-01753-4. PMC10791578 37081138

